# The effect of chronic soluble keratin supplementation in physically active individuals on body composition, blood parameters and cycling performance

**DOI:** 10.1186/s12970-018-0251-x

**Published:** 2018-09-27

**Authors:** Emma M. Crum, Yanita D. McLeay, Matthew J. Barnes, Stephen R. Stannard

**Affiliations:** School of Sport and Exercise, Massey University, Palmerston North, New Zealand

**Keywords:** Dietary protein, Exercise, Skeletal muscle, Ergogenic aid, Performance, DEXA

## Abstract

**Background:**

Keratins are structural, thiol-rich proteins, which comprise 90% of total poultry feather weight. Their favourable amino acid profile suggests the potential for use as a protein source and ergogenic aid for endurance athletes, following treatment to increase digestibility. This study investigated whether 4 weeks of soluble keratin (KER) consumption (0.8 g/kg bodyweight/day) by 15 endurance-trained males would have favourable effects on body composition, blood and cardiorespiratory variables, and cycling performance, compared to casein protein (CAS).

**Methods:**

Supplementation was randomized, blinded and balanced, with a minimum eight-week washout period between trials. An exercise test to measure oxygen consumption during submaximal and maximal cycling exercise was completed at the start at and end of each intervention. Anthropometric (DEXA) and blood measures were made prior to and following each intervention period.

**Results:**

Total body mass and percentage body fat did not change significantly (*p* > 0.05). However, a significantly greater increase in bone-free lean mass (LM) occurred with KER compared to CAS (0.88 kg vs 0.07 kg; *p* < 0.05). While no change in LM was evident for the trunk and arms, leg LM increased (0.45 ± 0.54 kg; *p* = 0.006) from baseline with KER. KER was not associated with changes in blood parameters, oxygen consumption, or exercise performance (*p* > 0.05).

**Conclusions:**

These data suggest that KER is not useful as an ergogenic aid for endurance athletes but may be a suitable protein supplement for maximizing increases in lean body mass.

## Background

Keratin proteins are tough, insoluble compounds, which are composed of a tight network of disulfide bonds, courtesy of their high thiol content [1]. Poultry feathers contain ~ 90% of their total weight as keratin, which gives the material its characteristic light but rigid structure [2]. In New Zealand, chickens are widely bred for use in commercial meat production (~ 82,000,000 birds annually) [3], but their feathers, which represent ~ 10% of total chicken weight, are indigestible to humans and other monogastrics and are therefore considered a significant biological waste product. To reduce costs of waste disposal, feather meal is commonly used as a component of animal feed [4]. Yet at dietary concentrations of > 8%, its consumption is associated with significant impairments in growth, weight gain and food intake, because, despite a favourable amino acid profile, its low digestibility renders much of the amino acids unavailable [5].

Conventional methods to improve digestibility have included hydrolysis, chemical and enzymatic processing treatments [1, 6]. However, during the cleaving of disulfide bonds, such methods also reduce the thiol content of keratin [6]. In addition, the oxidation processes involved in many previous methods of keratin extraction result in the production of peptides with strong off-tastes and odours, making the new product unpalatable [7].

Recently, a proprietary controlled hydrolysis process has been developed using poultry feathers, which produces a novel keratin product (KER) with a digestibility of ~ 83% in vivo, while maintaining a thiol component of ~ 87%. The procedure yields a protein powder which can be mixed with liquid and ingested as a drink, or formulated into a protein bar [7]. We have previously shown that ingestion of up to 40 g per day of soluble KER is safe and palatable when supplementing a balanced diet in humans [8]. Further, a rodent study by Wolber et al. (2016) indicated that the consumption of KER has potential benefits other than simply providing a cheap protein source (poultry feathers) [9]. Following the substitution of 50% of a casein (CAS) protein diet with KER in male Sprague–Dawley rats over a four-week period, blood profiles exhibited significant increases (~ 5%) in haematocrit (Hct) and haemoglobin content (Hb). In addition, dual-energy X-ray absorptiometry (DEXA) analysis suggested an improvement in lean to fat mass ratio and increased femoral bone mineral density (BMD) in the KER-supplemented rats compared to CAS alone. However, in this small scale, controlled trial, measures were made only at the end of the dietary intervention (i.e., no repeat measures), minimizing the statistical power.

Considering the improvements in erythrocyte and body composition parameters in rodents, KER has the potential to be used as an ergogenic supplement if the same changes can be induced in humans. The concentration of erythrocytes and Hb in whole blood are a significant limiting factor in oxygen (O_2_) transport and increases in these parameters are commonly targeted by performance enhancing interventions for endurance athletes [10]. A greater lean to fat mass composition is also advantageous for such individuals, who benefit from an improved power-to-weight ratio [11]. However, apart from the aforementioned studies, the literature is scarce regarding soluble KER supplementation and is non-existent in humans.

Therefore, using physically active healthy individuals, the purpose of this study was to compare the effects of 4 weeks of cycle training combined with KER or CAS supplementation on anthropometric, blood, cardiorespiratory and performance variables. Based on the observations of a study using a rodent model [9] and applying these to a repeated-measures cross-over design, we hypothesized that supplementation with KER would result in an increased lean to fat mass ratio, a more favourable blood profile, and consequently improved performance during cycling exercise when compared to supplementation with the same amount of CAS.

## Methods

### Participants

Fifteen trained male cyclists, aged 18–50 years, were recruited from the regional cycle and triathlon communities to participate in this study. The criteria for inclusion were regular participation in endurance cycling exercise, at least three times per week in the 3 months prior to commencement of the study, absence of any contraindications to exercise or other procedures involved in the research, or allergy/objection to the consumption of dairy or animal products. Due to the novelty of the intervention, the effect size difference (between the CAS and KER conditions) was unknown. It is acknowledged that a larger sample size would have provided greater power; however, due to the small pool of individuals who fit the participation criteria, this was not possible. The average age, mass, percentage body fat and VO_2max_ of the participants were 34 ± 11 years, 84.2 ± 13.9 kg, 21.5 ± 4.8% and 60.2 ± 8.7 ml.kg^− 1^ min^− 1^, respectively. Prior to participation, the participants were notified of all the potential risks and benefits associated with the study, and written and verbal consent were obtained. This study was approved by the University Human Ethics Committee, in accordance with the Declaration of Helsinki.

### Procedures

#### Design

**Fig. 1:**
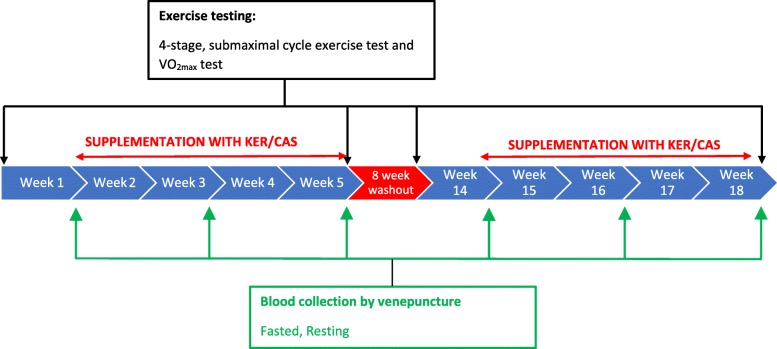
Schematic representation of the study design

The study used a randomized, blinded, balanced crossover design, with two four-week intervention periods (INT1: intervention 1; INT2: intervention 2) involving supplementation with either KER or a low-cysteine protein (“gold standard” sodium caseinate, CAS [9]). The interventions were separated by a minimum of 8 weeks to allow wash-out of any remaining traces of the supplement from the participant. At the beginning of each intervention, participants performed a protocol involving submaximal and maximal exercise on a cycle ergometer to measure O_2_ consumption and exercise performance. Supplementation began one-week after the exercise testing and continued for 4 weeks. At the end of the supplementation protocol, the exercise protocol was repeated. A diagram showing the timeline for one intervention is shown in Fig. 1.

#### Supplementation

During each intervention, participants consumed 0.8 g protein per kg bodyweight, per day (g.kg^− 1^d^− 1^), for 6 days per week, with one non-supplementation day each week. This was based off the recommended dietary intake for protein for males aged 18–70 of 0.84 g.kg^− 1^d^− 1^ [12]. Adding 0.8 g.kg^− 1^d^− 1^ with the assumption that the participants would habitually consume about 0.8–1.0 g.kg^− 1^d^− 1^ of protein provided between 1.6–2.0 g.kg^−1^d^−1^. The provision of the supplement in addition to regular protein intake ensured that participants did not miss out on any amino acids usually derived from a normal diet. Because CAS and KER differ in their protein fractions (CAS: 0.91; KER: 0.87), the amount of supplement given in each intervention was calculated as providing the desired protein content, rather than as a certain amount of supplement. The supplement was consumed as two protein bars (of two different flavours), and the remainder of the protein requirement in powder form, which was mixed with water to form a drink; the macronutrient contents of the supplements are provided in Table 1 and the amino acid profiles are provided in Table 2. Participants received 1 week’s worth of supplements at the beginning of each week during the supplementation period and filled out a weekly health/side effects questionnaire to monitor for any adverse effects of supplementation.

**Table 1:** Macronutrient contents of KER and CAS supplements

Supplement	Energy (kJ/100 g)	Protein (g/100 g)	Carbohydrate (g/100 g)	Fat (g/100 g)
KER powder	1536.9	69.0	17.7	1.7
CAS powder	1589.2	72.1	17.7	1.7
KER bar (banana)	1285.3	31.0	35.5	4.2
CAS bar (banana)	1306.1	32.2	35.5	4.2
KER bar (peanut)	1776.8	40.1	40.0	11.2
CAS bar (peanut)	1717.1	36.6	40.0	11.2

Note: Supplements were consumed as two bars + protein powder totalling 0.8 g.kg^−1^ was ingested per day

**Table 2:** Amino acid profile (grams of anhydrous amino acid per 100 g of protein) for keratin (Kerapro) and casein proteins

	Kerapro	Casein
Alanine	4.2	2.9
Arginine	7.0	3.5
Aspartic Acid	7.6	7
Cysteine^a^	6.5	0.7
Glutamic Acid	11.8	23.2
Glycine	6.3	1.7
Histidine	0.5	2.6
Isoleucine	5.3	4.1
Leucine	8.3	9.2
Lysine	1.2	7.5
Methionine	0.4	2.8
Phenylalanine	4.6	4.9
Proline	10.0	10.7
Serine	10.6	5.6
Threonine	4.9	4.2
Tyrosine	2.1	5.4
Valine	8.8	5.3

^a^primarily as cysteic acid

The novel KER supplement was supplied by Keraplast Research Ltd. (Christchurch, New Zealand). KER was produced from the feathers of white leghorn chickens via a patented minimal hydrolysis process (patent application number US 13/381,766), which is described in detail by Kelly and Marsh [7] . Briefly, this involved treating keratin in an oxidizing solution at a low pH, and then heating the mixture to oxidize cysteine (CYS) residues in the protein. Subsequently, the mixture was cooled, and a base was added to raise the pH of the solution to form a protein salt. The solution was dried and milled to form a protein powder. The CAS supplement was purchased from Tatua (Morrinsville, New Zealand), and formed into a protein powder and bar of similar taste and protein content to KER by Keraplast Research Ltd.

During the 3 weeks preceding, and throughout the duration of each trial, participants were asked to refrain from taking any form of supplement, including other protein powders or bars. Any prescribed supplements and/or medication were discussed with the researchers to deem whether they would have any potential effect on study measures.

#### Recording of diet and training

Starting from 1 week prior, until the end of the post-exercise testing in each intervention, participants recorded their daily training schedule using the online software, Training Peaks™ (Boulder, Colorado, USA) to detail the mode, duration and distance of any exercise they completed. The participants attended two one-hour cycle training sessions per week at the laboratory—a steady-state and a high intensity exercise session—to replicate common sessions in a cycling training programme. The power outputs prescribed for each protocol were determined by calculation of various percentages of the participant’s VO_2max_, as determined by their initial VO_2max_ test. The first session consisted of a 15-min progressive warm-up, followed by two 15-min steady state intervals at 80%VO_2max_; these were separated by 10 minutes at 60%VO_2max_ and were followed by a five-minute cool down. The second session involved a five-minute warm-up followed by intervals of 1 minute at 85%, 90%, 100% VO_2max_, 30 s at 130% VO_2max_, then 1 minute at 100%, 90%, and 85% VO_2max_. Light active recovery was given between intervals and a five-minute warm-down occurred at the end of the session. In additional to this prescribed exercise, participants completed their own training outside of the laboratory in their own time. To ensure training was consistent across trials, training recorded during INT1 was replicated in INT2. The exercise test at the start of the two intervention periods was used to confirm that fitness levels were the same prior to each bout of supplementation and indicated that the washout period had been sufficient. Dietary intake was also recorded during this time, using the online software, MyFitnessPal™ (Under Armour, Baltimore, Maryland). Participants input each item of food that they consumed during the required periods, and an associated database determined the daily caloric and macronutrient intakes. The participants were asked to follow a similar diet in both interventions; however, it was not feasible for the diet to be replicated exactly.

#### Body composition

At the beginning of each study, the weight of each participant was measured using a standard scale (Jadever, Taiwan; accurate to 0.01 kg). Body composition was evaluated prior to and following each trial using DEXA. This method is commonly accepted as the gold standard of body composition measurement and is used as a reference method for testing of other body composition systems [13]. After an overnight fast, subjects were scanned in a supine position and data on bone free lean mass (LM), fat mass (FM) and body fat percent (BF%) using DEXA software (APEX Software Version 4.5.3) were analysed. When differences were found, further analysis of body segments, i.e. leg, arm and trunk, was carried out to identify the location of these changes. To ensure neutrality, the technician operating and providing DEXA results was blinded to the treatments being investigated. To ensure consistency between measurements, participants completed this test wearing underwear and a thin robe and were positioned on the table using their first scan position as a reference.

#### Exercise protocols

All exercise testing was completed in a fasted condition, on an electronically-braked ergometer (Lode Excalibur, Groningen, The Netherlands) in thermoneutral conditions (18–20 °C). The four-stage, submaximal exercise test measured O_2_ consumption (VO_2_) at four submaximal workloads during cycling exercise. Following a five-minute warm-up at 100 W, participants completed four, seven-minute stages of increasing workload (e.g. 150, 200, 250, 300 W) with expired air being collected into Douglas Bags during the last minute of each stage and analysed for O_2_ and CO_2_ concentrations and volume. Following a five-minute active rest period, an incremental “ramp” protocol was used to determine VO_2max_. Power began at 100 W and increased linearly with time (25 W.min^− 1^). Participants cycled for as long as possible and verbal encouragement was given to elicit maximal effort. As the participant’s VO_2max_ was approached (as indicated by a change in breathing pattern), expired air was captured in Douglas bags until exhaustion. Analysis of Douglas bags was done using a calibrated gas analysing system (AD Instruments, Dunedin, New Zealand). The gas analyser was calibrated using gases of known concentration (15.01% O_2_, 5.01% CO_2_). Minute ventilation (V_E_) and concentrations of O_2_ and CO_2_ values were used to calculate the volume of inspired air (V_I_) using the Haldane transformation, where V_E_ was corrected for barometric pressure, ambient temperature and atmospheric water saturation. Subsequently, VO_2_ and expired CO_2_ (VCO_2_) could be determined and are reported as standard temperature and pressure dry (STPD). The respiratory exchange ratio (RER) was calculated using VCO_2_/VO_2_ and attainment of VO_2max_ was confirmed with RER ≥1.1. In accordance with Schlader ZJ, Raman A, Morton RH, Stannard SR and Mündel T [14], a relationship between steady-state workload and VO_2_ values was drawn through creation of a power curve and generation of a linear line equation y = mx + c, where m = gradient, x = power and c = start point. The equation was used to estimate power output at 65% of VO_2max._

#### Blood sampling

Fasted blood samples were taken at start, mid (2 weeks) and post-intervention time points. The pre-intervention blood sample was taken on the first day of supplementation, prior to ingestion of the supplement and the post-intervention sample was taken prior to the post-intervention exercise test (see Fig. 1). The sample was collected from a vein in the area of the antecubital fossa of either arm using a needle and vacutainer (BD, New Jersey, USA) in ethylenediaminetetraacetic acid (EDTA) vacutainers (~ 4 ml), and then immediately transferred to a fridge (~ 2 °C) and analysed within 48-h.

### Blood analysis

Whole blood samples were analysed by full blood count, using a fully-automated bench-top, five-part differential haematology analyser (LH 500, Beckman Coulter, Fullerton, CA, USA). The LH500 analyser has previously been validated against other such devices and with the haemoglobincyanide method, which is the reference method recommended by the International Committee for Standardization in Haematology [15, 16]. The analyser was calibrated prior to use as per the manufacturer’s recommendations and had shown acceptable performance on proficiency testing.

The specific erythrocyte parameters recorded were total erythrocyte count (RBC), haemoglobin (Hb) volume, haematocrit volume (Hct), mean cell volume (MCV) (the average volume of individual erythrocytes), mean cell haemoglobin concentration (MCH) and mean cell haemoglobin concentration relative to erythrocyte cell volume (MCHC). The total number of leukocytes (WBC) in whole blood was also measured.

### Statistical analysis

Statistical analyses were done using SPSS Statistics, Version 23(IBM Corporation, New York). Statistical significance was accepted when *p* ≤ 0.05, while a tendency was noted when *p* ≤ 0.1.

The expired gas variables taken during the four-stage submaximal protocol and VO_2max_ tests (VO_2_, VCO_2_) were analysed using three-way, repeated measures ANOVA to determine the main effects of, and interactions between, treatment (KER vs. CAS), exercise stage (1–4) and time point during the intervention (beginning vs. end). Power output at VO_2max_ (PO), body composition variables and dietary information were analysed using two-way, repeated measures ANOVA (treatment x time). Blood variables were also analysed using two-way repeated measures ANOVA (treatment x time) for significant differences between variables at pre, mid and post-intervention time points.

In all analyses, Mauchly’s Test of Sphericity was used, and if aspherity had been detected, the Greenhouse–Geiser correction would have been applied. However, this was not necessary in any case. Following each ANOVA, where significant main effects or interactions were observed, two-tailed paired t-tests with a Fisher’s Least Significant Difference (Fisher’s LSD) post-hoc analysis were used to identify the location of the significance. The effect sizes (ES) of significant interactions were calculated using Cohen’s d with the equation Cohen’s d = (M_2_ – M_1_)/SD_pooled_. In accordance with Cohen J [17], effect sizes were classified as small (0.1), medium (0.5) or large (0.8). Data is presented as mean ± standard deviation (SD) or mean change and 95% confidence interval (CI) as appropriate. Graphs were produced using Prism 6.0 (GraphPad Software, CA, USA).

## Results

### Side effects and blinding

No side effects of either condition were reported, aside from some anecdotal comments of occasional sulfur-smelling flatulence with KER. Participants were blinded to the contents of the supplements and only one participant was able to confidently guess which condition they were on.

### Diet and training

Participants attended all compulsory training sessions at the laboratory and reported that they followed the same training plan in both interventions. Five participants did not fully complete their food diary and were therefore excluded from the dietary analysis. The supplements (KER and CAS) provided to participants did not differ in energy, protein, fat or carbohydrate content (all *p* > 0.05). When the supplements were not included, repeated measures ANOVA revealed that there were no significant main effects of treatment on total energy intake from food during both four-week interventions (*F*_1,9_ = 0.137, *p* = 0.14). There was a main effect of treatment on protein intake, with subjects consuming significantly less protein during all 4 weeks (pooled) of KER (− 14.1 g, 95% CI, − 26.7, − 1.5, *F*_1,9_ = 6.37, *p* = 0.033, ES = 0.5).

### Body composition

When treatment was pooled (main effect of time) BF% and FM did not change during the 4 weeks (BF% *F*_1,14_ = 1.00, *p* = 0.33; FM, *F*_1,14_ = 0.14, *p* = 0.71). Similarly, there was no main effect of treatment (BF%, *F*_1,14_ = 0.022, *p* = 0.89; FM, *F*_1,14_ = 0.96, *p* = 0.34), and no treatment x time interaction was evident for either measurement (BF%, *F*_1,14_ = 1.47, *p* = 0.25, FM, *F*_1,14_ = 0.182, *p* = 0.68). There were no main effects on LM to FM ratio for either treatment (*F*_1,14_ = 0.113, *p* = 0.74) or time (*F*_1,14_ = 1.10, *p* = 0.31), nor was there an interaction effect (*F*_1,14_ = 1.82, *p* = 0.15). Changes in key anthropometric variables are outlined in Table 3 and segmented percentage changes in lean muscle mass are shown in Table 4.

**Table 3:** Key anthropometric variables, as measured via DEXA, after 4 weeks of cycling exercise and a diet supplemented with either KER or CAS protein

	Keratin		Casein	
	Pre	Post	Pre	Post
% BF	22.5 ± 4.3	22.2 ± 4.3	22.4 ± 4.7	22.4 ± 4.6
Lean mass (kg)	61.0 ± 8.2	62.0 ± 8.4^a^	61.7 ± 8.3	61.8 ± 8.1
Fat mass (kg)	19.1 ± 6.9	19.0 ± 6.6	19.2 ± 7.3	19.3 ± 7.2
Total Mass (kg)	85.1 ± 14.3	86.0 ± 14.1	86.3 ± 14.7	86.2 ± 14.3
Lean to fat ratio	3.4 ± 0.8	3.5 ± 0.9	3.5 ± 0.9	3.5 ± 0.9
Bone mineral density (g/cm^2^)	1.3 ± 0.1	1.3 ± 0.1	1.3 ± 0.1	1.3 ± 0.2

Note:^a^ denotes significantly different to Pre (*p* ≤ 0.05)

**Table 4:** Percentage changes in lean body mass of body segments following supplementation with KER and CAS

Body Segment	KER	CAS
Whole body	1.4 ± 2.3	0.2 ± 1.8
Left leg	2.1 ± 3.2^a^	0.25 ± 2.2
Right leg	1.9 ± 2.3^a^	− 0.64 ± 1.9
Left arm	1.4 ± 4.7	No change
Right arm	1.2 ± 3.5	No change
Trunk	0.92 ± 3.1	0.85 ± 2.3

^a^Indicates significantly different from CAS (*p* ≤ 0.05). Data are mean percentage change ± standard deviation

Although there was no main effect of either treatment (*F*_1,14_ = 0.879, *p* = 0.36) or time (*F*_1,14_ = 2.83, *p* = 0.12) on whole body LM, a significant treatment x time interaction was evident (*F*_1,14_ = 4.80, *p* = 0.043). Post hoc testing indicated that LM significantly increased from baseline during KER (+ 0.88 kg, 95% CI, 1.7, 0.12, *F*_1,14_ = 6.09, *p* < 0.03, ES = 0.1); however, there was no significant change in the CAS condition (+ 0.07 kg, 95% CI, − 0.61, 0.75, *F*_1,14_ = 0.046, *p* = 0.83). The analysis of changes in LM of body segments revealed no main effect of time (*F*_1,14_ = 2.27, *p* = 0.15) or treatment (*F*_1,14_ = 0.254, *p* = 0.62) and no treatment x time interaction (*F*_1,14_ = 0.079, *p* = 0.78) on trunk LM. Similarly, no change was seen in arm LM over time (*F*_1,14_ = 0.38, *p* = 0.55), or between treatments (*F*_1,14_ = 1.24, *p* = 0.28) and there were no treatment x time interaction effects (*F*_1,14_ = 3.86, *p* = 0.07). Significant treatment x time effects were found for leg LM (*F*_1,14_ = 15.2, *p =* 0.002) with LM increasing significantly from baseline with KER only (0.45 kg, 95% CI, 1,5, 7.4, *F*_1,14_ = 10.3, *p* = 0.006, ES = 0.2) while no change was evident for CAS (− 0.04 kg, 95% CI, − 2.5, 1.6, *F*_1,14_ = 0.222, *p* = 0.65). No main effects of time (*F*_1,14_ = 4.01, *p* = 0.07) or treatment (*F*_1,14_ = 2.14, *p* = 0.17) were found for leg LM.

### Blood variables

The blood parameter values for pre, mid and post-supplementation with CAS and KER are presented in Table 5. There were no significant main effects of treatment on RBC (*F*_1,14_ = 0.616, *p* = 0.45), Hb (*F*_1,14_ = 0.645, *p* = 0.44), Hct (*F*_1,14_ < 0.0001, *p* = 0.99), MCV (*F*_1,14_ = 0.716, *p* = 0.41), MCH (*F*_1,14_ = 0.182, *p* = 0.68), MCHC (*F*_1,14_ = 1.02, *p* = 0.33) or WBC (*F*_1,14_ = 1.44, *p* = 0.25) during the supplementation period. There were also no main effects of time on RBC (*F*_1,14_ = 2.37, *p* = 0.11), Hb (*F*_1,14_ = 0.683, *p* = 0.51), Hct (*F*_1,14_ = 1.86, *p* = 0.18), MCH (*F*_1,14_ = 0.086, *p* = 0.92), MCHC (*F*_1,14_ = 0.235, *p* = 0.79), MCV (*F*_1,14_ = 0.547, *p* = 0.59) or WBC (*F*_1,14_ = 0.680, *p* = 0.52).

**Table 5:** Blood variables measured at pre-, mid- and post-intervention time points during supplementation with either KER or CAS protein

	Keratin			Casein		
	Pre	Mid	Post	Pre	Mid	Post
RBC (× 10^12^/L)	5.0 ± 0.3	5.0 ± 0.3	5.0 ± 0.3	5.1 ± 0.3	5.0 ± 0.2	5.0 ± 0.3
Hct (%)	44.8 ± 2.0	43.9 ± 1.7	44.7 ± 2.0	44.7 ± 2.5	44.2 ± 1.7	44.5 ± 1.9
Hb (g.L^− 1^)	149 ± 6	147 ± 6	148 ± 7	148 ± 7	148 ± 5	150 ± 6
MCV (fL)	89.4 ± 2.8	88.9 ± 2.8	89.0 ± 2.3	88.8 ± 3.6	89.0 ± 3.3	88.5 ± 3.4
MCH (pg)	29.7 ± 1.1	29.7 ± 1.3	29.6 ± 1.3	29.6 ± 1.4	29.8 ± 1.1	29.7 ± 1.2
MCHC (g/L^− 1^)	332 ± 8	334 ± 12	332 ± 11	334 ± 15	335 ± 11	336 ± 9
WBC (× 10^9^/L)	5.7 ± 1.0	6.0 ± 1.3	5.7 ± 0.7	6.0 ± 1.2	6.1 ± 1.0	5.9 ± 1.0

### Cardiorespiratory data

The cardiorespiratory data is presented in Table 6. The four stages during the steady-state exercise test corresponded to ~ 42, 54, 65 and 73% of the participants’ initial VO_2max_.

**Table 6:** Cardiorespiratory variables, measured during the maximal oxygen consumption test

	VO_2_				VCO_2_			
	Keratin		Casein		Keratin		Casein	
	Pre	Post	Pre	Post	Pre	Post	Pre	Post
Stage-1	24.8 ± 3.2	27.9 ± 6.6	24.5 ± 4.1	26.5 ± 3.7	21.1 ± 3.0	22.4 ± 3.2	20.9 ± 4.1	22.6 ± 3.9
Stage-2	33.2 ± 4.0	34.7 ± 9.0	32.4 ± 4.1	32.8 ± 5.0	29.0 ± 3.9	28.0 ± 4.3	28.1 ± 4.1	28.5 ± 5.5
Stage-3	38.7 ± 4.4	41.1 ± 9.4	38.4 ± 4.9	39.5 ± 7.5	33.5 ± 4.3	33.7 ± 5.1	33.7 ± 5.2	34.0 ± 6.7
Stage-4	43.6 ± 5.5	45.5 ± 9.4	43.7 ± 5.3	45.7 ± 9.0	38.9 ± 5.5	38.0 ± 5.5	38.8 ± 5.1	38.3 ± 6.8
Exhaustion	59.8 ± 9.2	62.0 ± 9.5	60.7 ± 10.3	62.9 ± 10.7	63.9 ± 11.9	64.9 ± 10.8	65.0 ± 12.6	64.7 ± 11.1

There were no main effects of treatment on VO_2_ (*F*_1,13_ = 2.70, *p* = 0.13) or VCO_2_ (*F*_1,13_ = 0.12, *p* = 0.73) during the four-stage steady-state submaximal exercise or in VO_2max_ (*F*_1,12_ = 1.48, *p* = 0.25) or VCO_2max_ (*F*_1,12_ = 0.73, *p* = 0.411) during the VO_2max_ test. Further, there were no significant changes from pre to post-intervention in VO_2_ (*F*_1,13_ = 0.53, *p* = 0.48), VCO_2_ (*F*_1.13_ = 0.77, *p* = 0.52) or VCO_2max_ (*F*_1,12_ = 0.006, *p* = 0.94). There was a trend towards an increase in VO_2max_ post-intervention compared to pre-intervention (*F*_1,12_ = 3.67, *p* = 0.08).

There was a significant treatment x time interaction for VCO_2_ (*p* < 0.02), with post-hoc analysis showing greater values following KER compared to CAS, only in stage one of the exercise (+ 2.0 ± 0.7 ml.min.kg^− 1^, 95% CI, 0.2, 3.9, *F*_1,12_ = 5.8, *p* < 0.04, ES = 0.1).

As expected, there was also a highly significant increase in all pulmonary gas exchange and ventilatory parameters as the intensity of exercise increased (*F*_1,12_ = 137, *p* < 0.0001).

### Performance variables

**Fig. 2:**
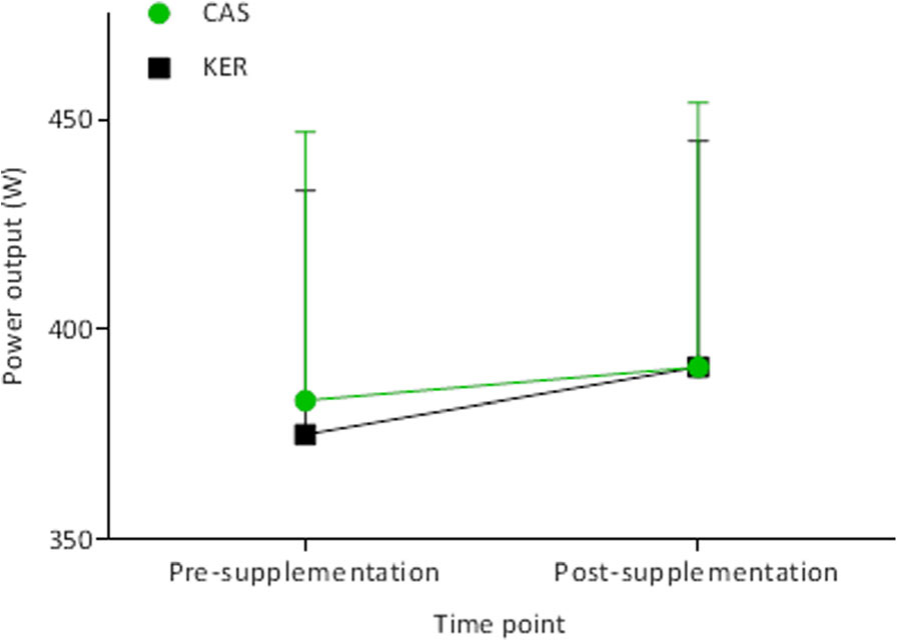
Power output at VO_2max_, pre- and post-supplementation with CAS and KER. Values are means ± SD

There was no significant main effect of treatment on the maximal PO achieved during the VO_2max_ test (*F*_1,14_ = 0.45, *p* = 0.51) and no significant treatment x time interaction (*p* = 0.23) (Fig. 2). Regardless of treatment, there was a significant increase in maximal PO at post- compared to pre-intervention (+ 12 W, 95% CI, 2, 22, *F*_1,14_ = 6.97, *p* = 0.02, ES = 0.2). Accordingly, PO at 65%VO_2max_ showed a strong trend towards being greater at post- compared to pre-exercise time-points (+ 7 W, 95% CI, − 0.1, 14, *F*_1,14_ = 4.44, *p* = 0.054, ES = 0.2), although this was not affected by treatment (*F*_1,14_ = 0.065, *p* = 0.80).

## Discussion

The purpose of this study was to compare the effects of supplementation with a cysteine-rich (KER) versus a low-cysteine (CAS) protein on anthropometric, blood, cardiorespiratory and performance variables during 4 weeks of aerobic exercise in physically fit individuals. While KER did result in a significant increase in LM, essentially in the lower limbs, when compared to supplementation with the same amount of CAS, there were no significant differences between treatments in any of the measured blood or cardiorespiratory parameters. Further, KER did not alter the maximal PO attained during a VO_2max_ test.

Although the beneficial effects of protein supplementation on LM and type I and II fibre cross sectional area during periods of resistance exercise have been extensively researched [18], little is known about the effects of protein supplementation on body composition during a period of endurance training. Cycling exercise has previously been shown to increase LM, particularly in the legs, however these observed changes have only been investigated after a prolonged period of training (i.e., twelve weeks [19]. Similarly, changes of up to 1 kg in whole body LM, as a result of resistance exercise and protein supplementation, are reported to occur after approximately twelve weeks [18]; given this magnitude of change and duration of resistance exercise training, the 1 kg increase in total lean mass and especially leg LM of approximately 0.5 kg is surprising—even more so when it is considered that the participants were already endurance cycling trained. The fact therefore, that significant increases in leg LM with KER, but not CAS, after only 4 weeks suggests that KER may have a potent effect on muscle protein synthesis, when paired with exercise. Whether this benefit is even greater with resistance exercise is certainly intriguing and worth further investigation.

Although significant effort was made to limit variation in exercise training and dietary intake between interventions, these factors could not be completely controlled, which presented a limitation to our results. Analyses of the supplemented and habitual diets, using self-reported rather than gold-standard weighed records, indicated no difference in total energy intake between conditions. Further, the protein and energy contents of the supplements were not different between conditions. However, there was a slight, but significantly greater protein intake, from non-supplemental food, during the CAS condition (+ 2.0 ± 0.7 ml.min.kg^− 1^). That LM increased in the KER condition whilst less protein was ingested is contrary to expectations. We are unable to explain this observation, though it emphasises the anabolic effect of KER. Only one participant claimed to be able to detect a difference in the KER and CAS supplements, so it is very unlikely that conscious behaviour was the cause of the different protein intake.

An explanation to these interesting LM observations may be twofold. Firstly, it is possible that supplementation with CAS is not conducive to maximizing LM during an aerobic exercise program when protein intake is increased above habitual levels. Few studies have identified the effects of CAS supplementation over a prolonged period of exercise; however Verdijk et al. [20] found no benefit of supplementing with 20 g of CAS, 10 g before and after resistance exercise, in the diet of older adults who already consumed enough protein. Similarly, no benefit of CAS supplementation in participants whose daily protein intake was already > 1.1 g/kg body weight, was evident here. This lack of change occurred irrespective of an increase in protein consumption of 2.14 ± 0.47 g/kg body weight and a significant increase in daily energy consumption. A second explanation is that the amino acid profile of soluble KER is more conducive, when superimposed on a habitual diet, to maximize lean body mass. Without prior published work it is difficult to provide a comparison. However a preceding rodent study [9] comparing CAS and KER supplementation suggested that KER may promote LM gain, although the results did not reach significance. There is an earlier published report that increasing the sulfur amino acid content (specifically CYS and methionine) above a CAS-based diet, in rodents at least, maximises weight gain [21]. That study, however, firstly did not partition between fat gain and lean mass, and secondly the processing of the raw keratin to produce the supplement used in the present study produces cysteic acid rather than CYS. Nevertheless, in relation to the latter, an increase in total thiol availability may somehow enable a faster LM accretion in muscles undergoing chronic contraction (exercise program). Clearly, mechanistic data is needed prior to any strong explanation being formed. Further, this study compared KER versus intake of a CAS supplement but did not include a non-supplemented condition. Therefore, we are unable to conclude whether supplementing with any type of protein is better than not supplementing at all.

The lack of change in blood parameters with KER in the current study is in contrast to the Wolber F et al. [9] study, which measured significant increases in Hct and Hb (both ~ 5%) in rats, following a four-week period of partial supplementation of a CAS diet with KER. Further, our results do not align with research involving another supplement with a similar thiol content, *N*-acetylcysteine (NAC), which has been associated with significant increases in plasma erythropoietin (EPO) (26%), Hct (10%), Hb (10%), MCV (12%) and MCH (3%) following 8 days of supplementation (1200 mg.d^− 1^) in untrained individuals [22]. It is possible that the previously-described effects of thiol supplementation on blood parameters may not occur in trained athletes, such as those who participated in the current study. Alternatively, previous research has found KER to have a digestibility of 78% in rats [9], but it is possible that the thiol content of the supplement was not digested sufficiently in our human participants. However, this is speculative, because digestibility was not measured in the current study.

Considering the lack of change in erythrocyte parameters, it is unsurprising that KER was not associated with any changes in VO_2_ or performance during the VO_2max_ test. Our hypothesis was based on the major role of erythrocytes in transporting O_2_ from the pulmonary system to the active muscles, and thus we assumed that any significant change in RBC, Hb or Hct would also lead to improved O_2_ transport. However, despite observing improvements in erythrocyte parameters following NAC supplementation, Zembron-Lacny et al. [22] did not measure any differences in VO_2max_ values obtained in a graded cycling test to exhaustion. Further, Kelly et al. [23] proposed that NAC supplementation (1800 mg) could improve VO_2_ through reducing respiratory muscle fatigue, thereby allowing faster O_2_ transfer into the blood stream. However, despite inducing an increase in the mean maximal inspiratory pressure during cycling at 85% VO_2max_, they showed no significant changes in VO_2_. Thus, based on previous literature and the current study, it appears that the provision of exogenous CYS, either as cysteic acid in KER, or as NAC, is not associated with improvements in O_2_ transport capacity.

Although VCO_2_ was generally not affected by treatment, there was an increase in values in stage 1 of the submaximal test only, which despite having a small effect size (ES = 0.1) was statistically significant. However, because this finding did not correspond to any of other measures, we are unable to determine whether this change was a true effect of KER or due to a type I or II error, particularly as the result of the small sample size.

The absence of change in maximal PO in the VO_2max_ in the current study with KER was also observed by Zembron-Lacny et al. [22] following 8 days supplementation with NAC in untrained individuals. Further, oral intake of thiol-containing supplements has not significantly improved performance in handgrip [24] or cycling time to exhaustion (TTE) protocols [25]. In addition, although Corn and Barstow [26] measured an increase in TTE with oral NAC supplementation (6000–7800 mg) during cycling at 80% of maximal PO, performance in tests at 90, 100 and 110% of maximal PO was not affected. Similarly Slattery et al. [27] demonstrated improvements in repeated sprint performance, but no change in total work or mean PO during a simulated cycling race by highly-trained athletes, following 9 days of NAC supplementation (1200 mg.d^− 1^). In contrast, Medved et al. [28] measured a large (~ 26%) increase in TTE during cycling at 90% VO_2max_, when a constant intravenous infusion of NAC was given during exercise to untrained but healthy individuals. Thus, it appears that thiol-containing supplements are most beneficial if taken in very large doses during exercise, a method which is impractical and disallowed in athletic competition. However, although this study observed no change in performance during a VO_2max_ test, it is possible that significant changes may have occurred with a different exercise protocol or with a larger sample size. Nevertheless, the absence of significant changes in the measured blood and cardiorespiratory variables do not indicate that KER has potential as an ergogenic aid during endurance cycling.

## Conclusions

Despite not inducing any significant changes in blood parameters, VO_2_ or performance measures, KER was well-tolerated by participants, and has the potential to be used as a high-protein supplement. Taken together with our previous study [8] we are confident that this food can be safely included in the diet of healthy adults for at least a 4 week period. Secondly, soluble KER may provide a more effective alternative to CAS as a protein supplement in those who wish to increase lean body mass in conjunction with an aerobic exercise program. These people potentially include older adults, athletes, and persons in the recovery phase of medical intervention or sickness where maximal rates of lean body mass accretion are necessary. The use of KER supplementation in strength-based athletes is thus an area of future research.

## Abbreviations

BF%: Body fat percentage
BMD: Bone mineral density
CAS: Sodium caseinate
CI: Confidence interval
CYS: Cysteine
DEXA: Dual X-ray absorptiometry
EDTA: Ethylenediaminetetraacetic acid
ES: Effect size
Fisher’s LSD: Fisher’s Least Significant Difference
FM: Fat mass
Hb: Haemoglobin
Hct: Haematocrit
INT: Intervention 2
INT1: Intervention 1
KER: Novel keratin product
LM: Lean mass
MCH: Mean cell haemoglobin concentration
MCHC: Mean cell haemoglobin concentration relative to erythrocyte cell volume
MCV: Mean cell volume
PO: Power output
RER: Respiratory exchange ratio
SD: Standard deviation
STPD: Standard temperature and pressure dry
TTE: Time to exhaustion
VCO_2_: Volume of expired carbon dioxide
V_E_: Minute ventilation
V_I_: Volume of inspired air
VO_2_: Rate of oxygen consumption
VO_2max_: Maximal rate of oxygen consumption
